# Corneal Collagen Cross-Linking with and without Epithelial Removal: A Contralateral Study with 0.5% Hypotonic Riboflavin Solution

**DOI:** 10.1155/2014/619398

**Published:** 2014-06-22

**Authors:** Aleksandar Stojanovic, Wen Zhou, Tor Paaske Utheim

**Affiliations:** ^1^SynsLaser Kirurgi AS, 9007 Tromsø, Troms, Norway; ^2^Eye Department, University Hospital North Norway, Sykehusveien 38, 9019 Tromsø, Troms, Norway; ^3^Department of Medical Biochemistry, Oslo University Hospital, 0450 Oslo, Norway

## Abstract

*Purpose*. Our main purpose was to compare safety and efficacy in the treatment of progressive keratoconus with “epithelium-on” and “epithelium-off” corneal collagen cross-linking (CXL). Our secondary purpose was to evaluate efficacy of CXL when hypotonic 0.5% riboflavin is used as photosensitizer. *Methods*. One eye of 20 patients with bilateral progressive keratoconus was randomly treated for “epithelium-on” CXL (group 1) while the fellow eye underwent “epithelium-off” CXL (group 2). Hypotonic 0.5% riboflavin was used in both groups. Visual acuity, refraction, corneal topography, and wavefront aberrometry were evaluated at baseline and after 1, 6, and 12 months. Specular microscopy was performed on 10 patients preoperatively and after 12 months. Postoperative pain was evaluated using a patient questionnaire. *Results*. Uncorrected and corrected distance visual acuity improved significantly in both groups. Refraction, topography, and aberrometry showed nonsignificant changes from the preoperative status throughout the 12-month follow-up in both groups. Moreover, the outcomes between the groups were comparable at all follow-up points. Endothelial cell-count was stable. Postoperative pain length was shorter in group 1 (*P* < 0.001). *Conclusion*. “Epithelium-on” and “epithelium-off” CXL using hypotonic 0.5% riboflavin were equally safe and effective in stabilization of keratoconus. Topography and aberrometry outcomes in both groups failed to show any significant improvements. This study is registered at ClinicalTrials.gov: NCT01181219.

## 1. Introduction

Reaction between riboflavin (vitamin B2), oxygen, and UV-A light is utilized in corneal collagen cross-linking (CXL) for creation of additional covalent bonds between the corneal stromal collagen fibers [1, 2]. Proper penetration of riboflavin within the stroma is essential for CXL because the riboflavin acts both as a photosensitizer leading to collagen cross-linking and as a protective agent shielding the deeper ocular structures from UV-A irradiation [3, 4]. However, the corneal epithelium is impermeable to compounds such as riboflavin, whose molecular weight is greater than 100 Da [5]. Therefore, epithelial debridement is normally performed in standard “epithelium-off” CXL to allow a dextran-based 0.1% isotonic riboflavin solution to penetrate the corneal stroma [3].

Several laboratory and clinical studies indicate that standard CXL is effective in increasing the biomechanical rigidity of the cornea and stopping the progression of keratoconus [2, 6–11]. Standard CXL, however, may lead to serious complications like postoperative infection [12], stromal haze [13], and corneal melting [14]. Hence, a CXL technique that does not require epithelial removal may be preferable to increase the safety of the procedure. For this reason, and for the assumed increase in patient comfort, transepithelial CXL (“epithelium-on” CXL) was proposed [15]. Thereafter, several approaches have been pursued to solve the major limitation of the “epithelium-on” CXL—an inadequate and inhomogeneous riboflavin penetration [5]. These include partial grid-like pattern deepithelialization [16], excimer laser superficial epithelial removal [17], the replacement of the isotonic by hypotonic riboflavin solution [18], and chemical enhancers such as benzalkonium chloride (BAC) [19], trometamol and ethylenediaminetetraacetic acid (EDTA) [20], tetracaine [21], and ethanol [16].

The current study utilizes a multifactorial approach to enhance the riboflavin penetration by employing: (1) BAC-containing local preoperative medication; (2) hypotonic riboflavin solution without dextran; (3) increased concentration of riboflavin (0.5%); and (4) prolongation of the riboflavin-induction time until objective verification of the stromal saturation is confirmed. This protocol has proved to be safe and effective in treatment of progressive keratoconus in a retrospective study by Stojanovic et al. [22]. The main aim of the current study was to evaluate “epithelium-on” and “epithelium-off” corneal CXL by comparing their safety and efficacy in eyes with progressive keratoconus using a randomized, contralateral, prospective design with the proposed treatment protocol. The secondary aim was to evaluate the efficacy of a hypotonic 0.5% riboflavin solution, which has only been reported in one previous study [22].

## 2. Patients and Methods

The treatments were performed at The Eye Department of the University Hospital North Norway, Tromsø, Norway. Inclusion criteria were as follows: (1) patients with documented progression of keratoconus during the last 12 months before treatment (increase of astigmatism or myopia by 1.00 D or increase in average Sim *K* by 1.50 D); (2) minimum corneal thickness of no less than 400 *μ*m at the thinnest point measured by Scheimpflug-based corneal topo-/tomography (Precisio, iVIS Technology, Taranto, Italy); and (3) Amsler-Krumeich keratoconus classification stages II to III. Exclusion criteria were as follows: (1) history of herpes virus keratitis; (2) severe dry eye; (3) concurrent corneal infections; (4) previous ocular surgery; and (5) hard contact lens wear ≤4 weeks before the baseline examination.

### 2.1. Pre- and Postoperative Examinations

Pre- and postoperative examinations consisted of slit lamp biomicroscopy, tonometry (Icare tonometer, Revenio Group Corporation, Helsinki, Finland), Precisio topo-/tomography, Placido disk-based topography and wavefront aberrometry (OPD-Scan II, Nidek. Co., Ltd. Aichi, Japan), uncorrected (UDVA) and corrected (CDVA) distance visual acuity measurement, and manifest refraction measurement (Nidek RT 2100 system, Nidek Co. Ltd., Aichi, Japan). The patients were examined preoperatively and 1, 6, and 12 months postoperatively. The verbal rating scale [23] was used in patients' evaluation of pain. The patients were asked to denote the postoperative pain intensity with a list of adjectives; these adjectives were assigned numbers from 0 to 5 (Table 1). The patients were also questioned on how many hours after the surgery the pain occurred, when it was most intense, and which of the two eyes was considered the most comfortable.

The regional ethics committee (REK Nord) approved the study. The research complied with the tenets of the Declaration of Helsinki. Written informed consent was obtained from each participant before examination.

### 2.2. Surgical Technique

One eye of the patient was randomly chosen to be treated with “epithelium-on” CXL and the fellow eye was treated with “epithelium-off” CXL. For each patient, the eye with the best CDVA was determined as the “best eye.” Blocked randomization was used to ensure that each group had an equal number of “best eyes.” The treatments of two eyes were performed 1 to 6 months apart. To reduce the risk for UV-exposure of the retroiridal eye structures, miosis was induced by application of two drops of pilocarpine 2% (Pilokarpin, Ophtha AS, Norway) in both groups.

The “epithelium-on” CXL was performed without epithelial debridement. Two drops of local anesthetic proparacaine 0.5% (Alcaine, Alcon Norway AS), preserved by 0.001% BAC, and two drops of local antibiotic gentamycin 0.3% (Garamycin, Schering-Plough AS, Norway), preserved by 0.005% BAC, were applied to the cornea, one drop every minute for the initial five minutes. Thereafter, two drops of proparacaine and two drops of hypotonic 0.5% aqueous riboflavin solution without dextran (Vitamin B2; Streuli, Uznach, Switzerland) were applied alternating every 30 seconds until the riboflavin saturation was verified by the slit-lamp inspection of the cornea and by determination of presence of riboflavin flare in the anterior chamber.

In the “epithelium-off” group, the epithelium was removed in a diameter of 8 mm with an Amoils-epithelial scrubber (Innovative Excimer Solutions Inc, Toronto, Canada). Two drops of proparacaine and two drops of hypotonic 0.5% aqueous riboflavin solution without dextran were applied alternating every 30 seconds until the riboflavin saturation was verified in the same way as group 1.

For both groups, the initial slit-lamp saturation evaluation was performed 15 minutes after the first application of riboflavin and repeatedly every five minutes until the saturation was confirmed. During the premedication and riboflavin induction, the patient was in supine position with eye speculum inserted. In group 1, irrigation with isotonic balanced salt solution (BSS) was performed before the UVA irradiation in order to avoid UVA-attenuation by the shielding effect of riboflavin covering the epithelium. Ring-shaped Merocel shield k20-5021 (Katena Products, Inc. Denville, NJ) was used to protect the limbal region and its stem cells from UVA radiation.

The cornea was subjected to UVA radiation with a wavelength of 365 nm at a working distance of 5 cm for 30 minutes. The UV-X lamp (IROC AG, Zürich, Switzerland) provided an irradiance of 3 mW/cm within a circular diameter of 9 mm. During the irradiation, BSS was applied every three minutes and proparacaine drops were added as needed.

After the UVA irradiation, two drops of atropine 1% (Atropine minims, Chauvin, England) and 2 drops of gentamycin were applied. The cornea was protected with a soft bandage contact lens for 12–18 hours for group 1 and for one week for group 2. Instructions were given to apply a mixture of 0.1% dexamethasone and 0.5% chloramphenicol (Spersadex med Kloramfenikol, Novartis, Norway) eye drops four times daily for one week, as well as to use artificial tears as needed.

In a subgroup of 10 patients (20 eyes), specular microscopy was performed with Konan CellCheck XL (Konan Medical, Irvine, CA) preoperatively and 12 months postoperatively to obtain the endothelial cell count.

### 2.3. Statistical Analysis

All visual acuity values were recorded as Snellen values and converted to LogMAR for statistical analyses. Statistical analysis was performed using SPSS 17.0 software. The difference in patients' subjective pain score between groups was analyzed by Wilcoxon signed ranks test. The distribution of all the other data was tested by Kolmogorov-Smirnov test. The paired *t*-test was used to assess differences at each follow-up point between the two groups as well as the changes of pre- and postoperative data within the groups. A *P* < 0.05 was considered statistically significant.

## 3. Results

All of the 20 patients recruited in the study were available for evaluation. The mean age of the 17 men and 3 women was 29.5 years (range 19 to 51 years). All measured data had normal distribution.

### 3.1. Pain Evaluation

Table 2 shows patients' subjective pain evaluation. There was no significant difference in the pain score; however, the most intense pain occurred earlier and had a shorter duration in group 1. Thirteen of 20 patients stated preference for “epithelium-on” CXL, 5 patients preferred “epithelium-off” CXL, and 2 patients evaluated the two techniques as equally uncomfortable.

### 3.2. Visual Acuity and Refraction

**Figure 1:**
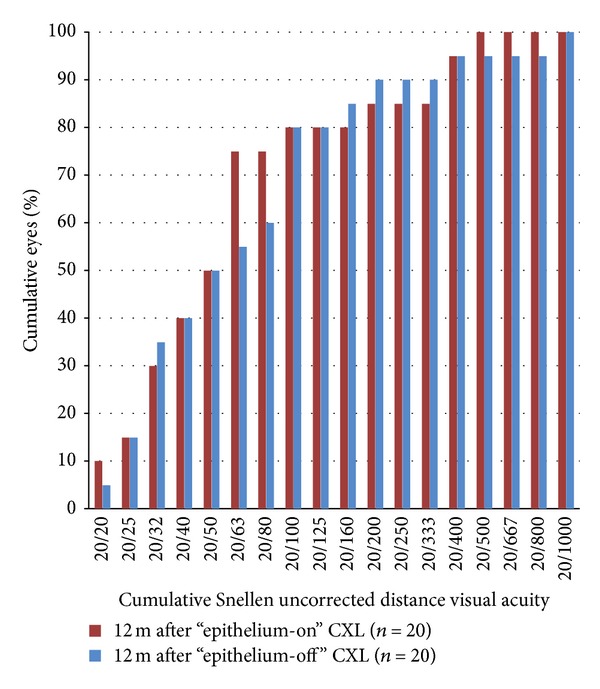
UDVA 12 months after “epithelium-on” and “epithelium-off” CXL.

**Figure 2:**
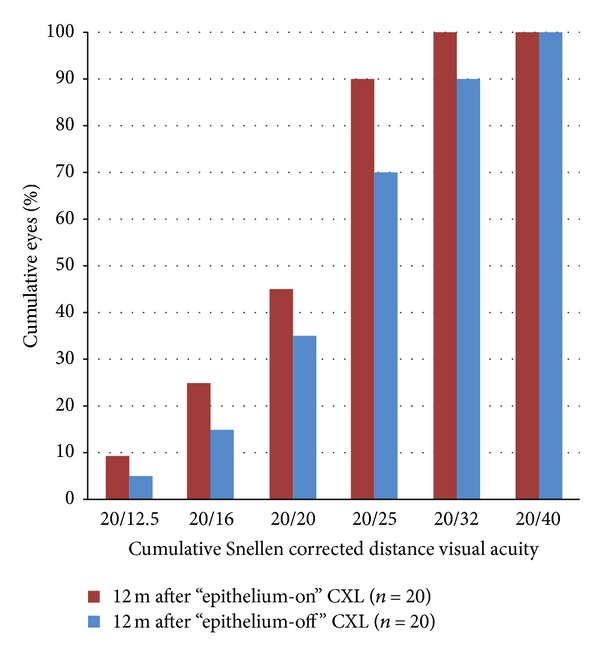
CDVA 12 months after “epithelium-on” and “epithelium-off” CXL.

**Figure 3:**
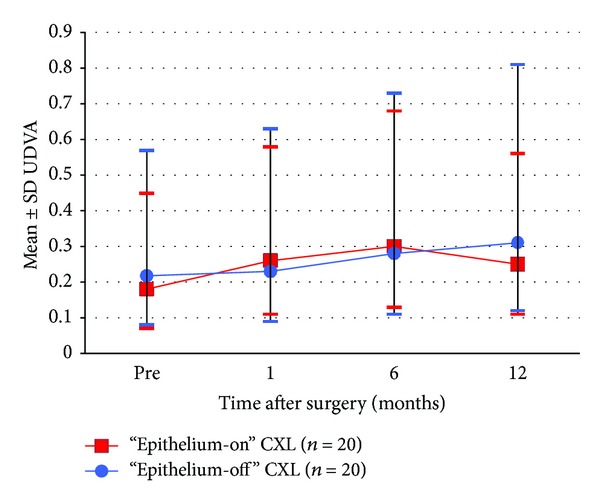
Development in UDVA pre- and 1–12 months postoperative.

**Figure 4:**
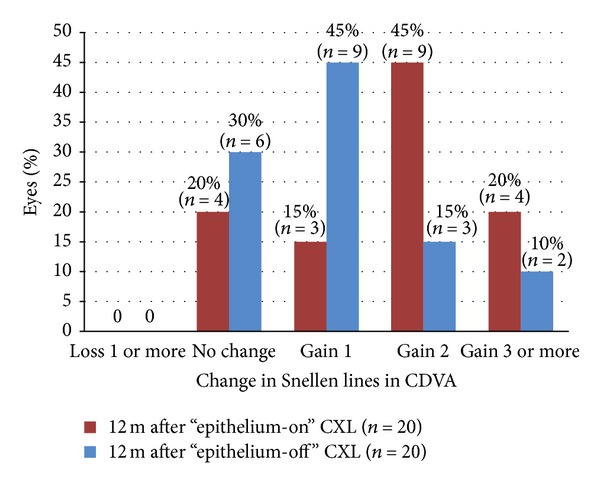
Change in Snellen lines in CDVA 12 months after “epithelium-on” and epithelium-off” CXL.

**Figure 5:**
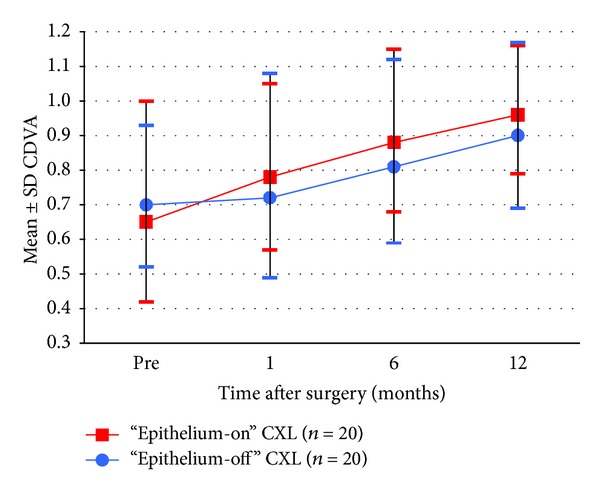
Development in CDVA pre- and 1–12 months postoperative.

**Figure 6:**
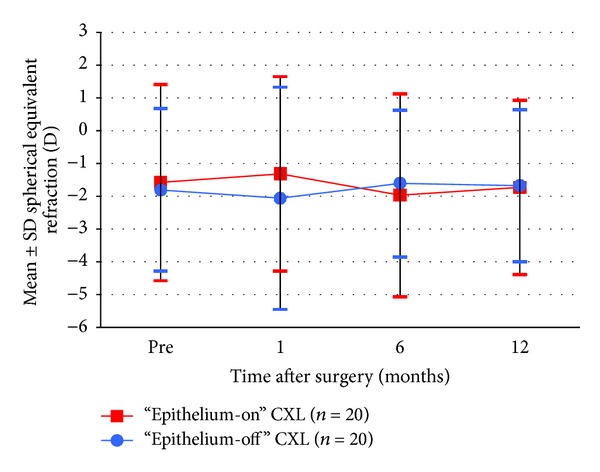
Stability of SE 1–12 months after “epithelium-on” and epithelium-off” CXL.

**Figure 7:**
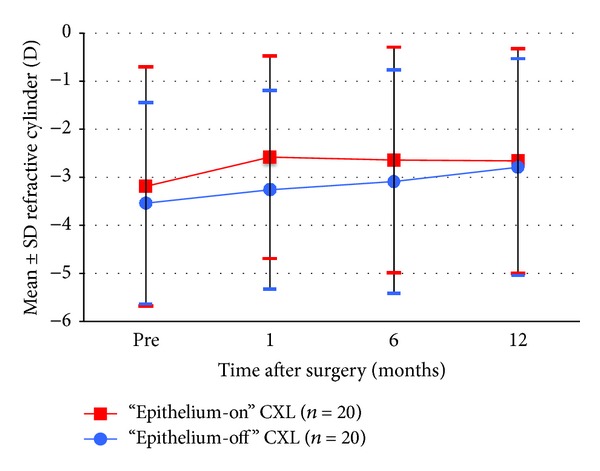
Stability of refractive cylinder 1–12 months after “epithelium-on” and epithelium-off” CXL.

Figures 1, 2, 3, 4, 5, 6, and 7 and Table 3 show the visual acuity and refraction pre- and postoperatively for both groups. None of the eyes lost lines of CDVA, while 65% of the eyes in group 1 and 25% of the eyes in group 2 gained 2 or more lines. Safety index for group 1 and group 2 was 1.50 and 1.29, respectively. Mean spherical equivalent refraction and refractive cylinder remained stable compared to preoperative status throughout the 12-month follow-up in both groups. No significant difference was found in visual acuity or refraction measurements between the two groups at any follow-up point.

### 3.3. Corneal Topography and Wavefront Aberrations

Table 4 shows pre- and postoperative topography features and wavefront aberrations. Scheimpflug topography data showed nonsignificant change in irregularity index (IRI) throughout the 12-month follow-up in both groups. Posterior corneal elevation increased nonsignificantly throughout the 12-month follow-up in both groups.

**Figure 8:**
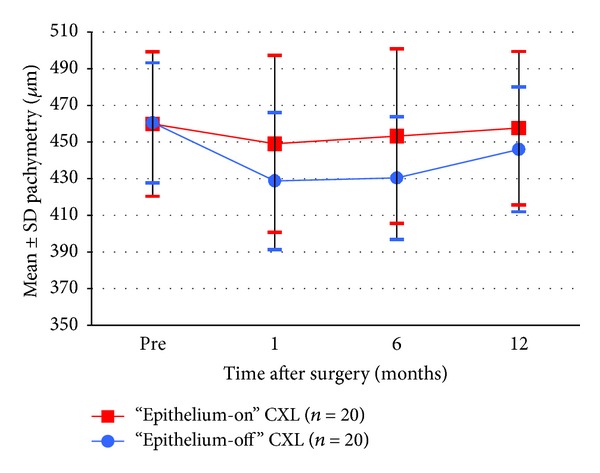
Change in pachymetry 1–12 months after “epithelium-on” and “epithelium-off” CXL.

Scheimpflug tomography-measured pachymetry in Figure 8 shows a decrease in thickness at the 1-month follow-up and a gradual increase in both groups thereafter. However, the thickness of corneas from group 1 increased to the preoperative level by the 12-month follow-up (*P* = 0.273), while the thickness of corneas from group 2 remained thinner than that measured preoperatively (*P* = 0.019).

The Placido-topography data regarding curvature along the steepest meridian (sim-*K*1), the flattest meridian (sim-*K*2) (both measured within the central 3 mm zone), and at the location of the cone (max-*K*) did not show significant changes in either of the two groups throughout the 12-month follow-up.

Higher order aberrations measured within the central 5 mm zone did not show significant changes either in RMS or in odd-order (S3 + 5 + 7) throughout the 12-month follow-up in both groups.

No significant differences were found in the measurements of topography features or wavefront aberrations between the two groups at any follow-up point.

In the subgroup of 20 eyes of 10 patients where specular microscopy was available, endothelial cell-count changed from 2544.7 ± 330.3 and 2577.6 ± 265.7 cells/mm^2^ to 2532.4 ± 325.3 and 2580.6 ± 261.1 cells/mm^2^ for groups 1 and 2, respectively. The change within each group was neither statistically significant (*P* = 0.101 and 0.725 for groups 1 and 2, resp.) nor was the difference between the groups (*P* = 0.499 and 0.349 for the preoperative difference between groups and 12-month postoperative difference between groups, resp.).

No postoperative complications were recorded in the current population.

## 4. Discussion

The “standard” CXL-protocol described by Wollensak and colleagues includes mechanical removal of the corneal epithelium in a diameter of 7 mm and use of 0.1% isotonic riboflavin solution in 20% dextran as a photosensitizer [3]. This protocol proved to be effective in the stabilization of keratoconus and improved the refractive and topographic features in most cases. Henriquez and coworkers [11] showed that the “standard” CXL method reduced spherical equivalent (by 2.25 D), maximum and minimum keratometry values (by 2.66 D and 1.61 D, resp.), and anterior and posterior elevation, which was assumed to be the cause of UDVA improvement. Vinciguerra et al. reported the use of “standard” CXL-protocol in stage III keratoconus [9]. The procedure led to UDVA and CDVA improvement and a decrease of the steep Sim *K* from 50.37 D to 44.21 D, as well as reduction of corneal asymmetry and spherical aberration. Epithelial removal has also been attempted by use of excimer laser; either via lamellar phototherapeutic keratectomy (PTK) [24], topography guided [25], or noncustomized PRK [26]. In addition to gentle epithelial removal, all three options showed stabilizing CXL effect and certain visual improvements due to better corneal optics.

“Epithelium-on” CXL was introduced [15] in 2010 with the rationale of eliminating the complications ascribed to deepithelialization; such as decreasing postoperative pain, making CXL possible on thin corneas and in less cooperative patients, increasing vision during the initial postoperative period, and lowering requirements for a sterile environment. Wollensak and Iomdina found a significant increase in corneal rigidity after “epithelium-on” CXL. However, the effect was estimated to be only one fifth of “epithelium-off” CXL [27]. Using 0.1% riboflavin solution containing trometamol (Tris-hydroxymethyl aminomethane) and sodium EDTA, Filippello et al. [20] showed stabilization of keratoconus with improvement of all visual, topographic (steep Sim *K* decreased from 51.02 D to 48.05 D; apical *K* from 59.12 D to 57.95 D), and aberrometric parameters (RMS higher order aberrations decreased from 4.68 *μ*m to 3.93 *μ*m, coma form 2.21 *μ*m to 2.11 *μ*m, and spherical aberration form 0.98 *μ*m to 0.73 *μ*m). The efficacy of the “epithelium-on” CXL has also been assessed* in vivo* in rabbits by comparing hypoosmolar 0.1% riboflavin solution containing BAC 0.02% and BAC 0.04% with isotonic unpreserved 0.1% riboflavin in dextran solution [19]. Stress-strain measurements showed that treatment with hypoosmolar riboflavin induced a sufficient increase in epithelial permeability to allow passage of riboflavin, resulting in increased corneal stiffening similar to that with “epithelium-off” CXL using isotonic solution. However, Koppen et al. [28] showed a statistically significant progressive increase of Scheimpflug-derived maximum *K* and decreased pachymetry throughout their clinical study, concluding that the “epithelium-on” CXL was less efficient than the “epithelium-off” CXL, despite the stabilized Placido-ring derived topographic features. Similar results were found in another clinical study [29] on “epithelium-on” CXL that used a modified protocol with a prolonged riboflavin-induction time of 4 hours and eye drops containing gentamicin, EDTA, and BAC. Caporossi and colleagues [30] demonstrated that keratoconus in young patients with rapid progression was relatively stable only in the first 12 months after “epithelium-on” CXL and returned to preoperative status by 24 months. They concluded that “epithelium-on” CXL can be recommended for patients with thin corneas (with the thinnest point less than 400 *μ*m) and in patients older than 26 years with slowly progressive keratoconus; but not for pediatric patients 18 years or younger whose cornea was 400 *μ*m or higher at the thinnest point. A retrospective study using a similar protocol to that employed here, with a wide range in population, concluded that the “epithelium-on” CXL was effective and safe in halting progressive keratoconus and to some extent in improving the corneal shape [22].

Baiocchi et al. [5] used 0.1% riboflavin-dextran 20% solution to soak the human corneas* in vitro*. The authors demonstrated that stromal concentrations of riboflavin increased with exposure only if the epithelium was removed, which was ascribed to the impermeability of the corneal epithelium for substances with molecular weight greater than 100 Da (riboflavin has a molecular weight of 338 Da). Consequently, the clinical studies applying the same or only slightly modified “epithelium-off” CXL protocol without removal of the epithelium showed very limited or no efficacy due to the insufficient and inhomogeneous transepithelial riboflavin diffusion into the corneal stroma. Hence, the use of the “epithelium-on” CXL requires a protocol modification to assure that the proper amount of riboflavin reaches the stroma before UVA-irradiation commences. Reported clinical studies with “epithelium-on” CXL, where efficacy in treatment of keratoconus was confirmed [20, 28, 29], all used modified versions of the standard “epithelium-off” CXL-protocol.

In the current study, a multifactorial approach described earlier was applied [22]. First, chemical disruption of the epithelial tight junctions was attempted by application of several tensio-active substances including BAC, tetracaine, and gentamicin [31]. Secondly, hypotonic riboflavin solution without use of dextran was used to increase the permeability of the corneal epithelium [32]. Wollensak et al. [33] found that hypotonic riboflavin solution also helps increase riboflavin penetration into the stroma. Thirdly, the concentration of riboflavin in our hypotonic solution was increased from 0.1% to 0.5% to increase the concentration gradient across the epithelium, with an aim to enhance its penetration and achieve higher UVA absorption [33]. The absorption coefficient of riboflavin has been found to linearly correlate with concentration up to 0.5% [34, 35]. Moreover, Bottos et al. found improved penetration (i.e., after 30 minutes) through intact epithelium in rabbit corneas with 1% compared to 0.1% riboflavin [36], yielding a stromal riboflavin concentration (expressed as UV-absorption coefficient) of 20.27/cm and 18.50/cm, respectively. In the current study, we confirmed riboflavin saturation by slit-lamp inspection of the cornea and by the presence of riboflavin flare in the anterior chamber before UVA-irradiation was attempted, without any time limits on the induction time (riboflavin saturation time was 38.2 minutes for group 1 and 19.8 minutes for group 2).

Investigation of potential differences in CXL-efficacy in treatment of keratoconus with different protocols is a formidable task, since the baseline of the treated population can vary significantly according to the severity of disease. Furthermore, it is hampered by differences in cone localization and shape as well as variations in population age. We used a contralateral design with blocked randomization to minimize the bias of the inhomogeneous baseline when comparing the “epithelium-on” and the “epithelium-off” CXL. To minimize possible confounding factors, we used hypotonic 0.5% riboflavin solution in both “epithelium-off” and “epithelium-on” CXL groups, although its efficacy in the “epithelium-off” protocol has not been shown elsewhere.

The pain score reported by the two groups in the current study showed no significant difference. The results suggest that even without deepithelialization, pain or discomfort may be attributed to apoptosis of keratocytes and damage of anterior stromal nerve fibers caused by toxic effects of CXL, as reported in several studies [6, 30, 37]. Corneal denervation could theoretically generate dry eye-related problems due to the decreased blinking rate and increased tear evaporation and exposure of corneal surface. However, Kontadakis et al. [37] reported no significant change in Schirmer's *I* test and tear film break-up time after 1 month post CXL, and Taneri et al. [38] found pathologic staining with fluorescein and Rose Bengal, as well as tear film height at 3 and 6 months after CXL, to be comparable to preoperative measurements. These findings revealed that dry eye does not seem to be a significant complication after CXL in patients with keratoconus. This is in accordance with the current study, where no patient complained about symptoms of dry eye after 1 month postoperatively.

The results of the current study showed visual improvements and no progression of keratoconus after the treatment and throughout the 12-month follow-up period in both groups. No significant difference between the groups was observed at any point. However, the effect of CXL, as normally estimated by corneal topography- and wavefront aberrometry-changes, was lower than previously reported, for both groups. Neither Sim *K*-values, maximum *K*-values, nor higher order aberrations were reduced as previously shown for both “epithelium-off” CXL [9, 11] and “epithelium-on” CXL [20]. As all previous reports applied 0.1% riboflavin, we speculate that use of 0.5% riboflavin solution may have caused the decrease in effect of CXL in the current study. One possible rationale for this is the increased use of oxygen by riboflavin due to its higher concentration in the stroma. Richoz et al. [39] recently revealed that the removal of oxygen after 30 seconds of irradiation essentially halts the photopolymerization process in CXL. They concluded that CXL requires oxygen in sufficient quantities to participate in the reaction. It is conceivable that when CXL is performed with 0.1% riboflavin, the supply of oxygen balances its consumption and allows the CXL process to continue. In contrast, the increased riboflavin concentration in the stroma, due to the use of hypotonic 0.5% solution, may have led to quicker oxygen consumption and therefore a reduction in the efficiency of CXL. Additionally, according to Larrea and associates [40], the diffusion factor for oxygen in human corneal stroma is calculated to be 2.81 × 10^−5 ^cm^2^/s, while in water it is 2.10 × 10^−5 ^cm^2^/s, implying that corneal hydration caused by the hypotonic solution may have led to slower oxygen transportation. This may have contributed to a prolonged state of corneal hypoxia, bringing the process of CXL to an early halt. Finally, it is possible that the retrospective study, where 0.5% riboflavin was used in a similar protocol, showed a somewhat better effect “improving the corneal shape to some extent” [22] due to the difference in the position of the patient during the riboflavin induction. Riboflavin induction performed with the patient in a supine position in the current study, as opposed to a sitting position in the retrospective study, may have led to a higher riboflavin concentration in the corneal stroma in the latter, resulting in higher consumption of oxygen and halting of the CXL reaction. This may explain the somewhat better effect in terms of corneal shape in the retrospective case study.

Epithelial absorption/filtering of UVA radiation that could potentially lead to less energy delivery to riboflavin saturated stroma has been stated as an argument against the use of “epithelium-on” CXL. However, previous studies draw different conclusions. A study by Baiocchi et al. [5] claims that human corneal epithelium and the underlying basement membrane naturally absorb 30% to 33% of UVA radiation (400 to 350 nm), while other studies showed that the epithelial UV absorption occurs only with wavelengths lower than 310 nm [41]. Bottós et al. [42] showed that the porcine corneal epithelium reduces the effectiveness of CXL by preventing the penetration of the drug, not by limiting the UVA transmittance. We assumed UV absorption of riboflavin within the epithelium to be low for two reasons. First, the epithelial cells are hydrophobic and do not absorb riboflavin and, second, the epithelial interstitial space is of negligible volume. To minimize the potential attenuation of UV radiation, we washed the corneas in the “epithelium-on” CXL group with BSS before the procedure and did not add any riboflavin to the cornea during the treatment.

## 5. Conclusion

The current study showed no difference in safety and efficacy between the “epithelium-on” and the “epithelium-off” CXL using a protocol that ensures corneal saturation with 0.5% hypotonic riboflavin solution. However, efficacy in reversing topography features of keratoconus with use of 0.5% hypotonic riboflavin seems to be lower compared to the reported results with “standard” 0.1% riboflavin.

## Figures and Tables

**Table 1:** Verbal rating scale for pain after CXL.

Assigned number	Intensity of the pain	Description of the pain
0	No pain	No pain or discomfort
1	Very little	Mild discomfort (foreign body sensation, dry eye…), no pain
2	Little	Mild pain
3	Intermediate	Moderate pain, released by closing the eyes or by artificial tears
4	Much	Severe pain (relieved by use of oral analgetics)
5	Very much	Unbearable pain (must use oral analgetics and local anesthetic drops)

**Table 2:** Pain evaluation.

	Pain score	Time point of the most intense pain (hour)	Pain length (hour)	Preference
“Epithelium-on” CXL	3.03 ± 0.73	3.78 ± 1.67	11.63 ± 5.89	13
“Epithelium-off” CXL	3.33 ± 0.38	6.25 ± 3.38	33.90 ± 23.76	5
*P* value	0.3765	0.002	0.000	—

**Table 3:** Changes in visual acuity and refraction during 12-month follow-up.

Parameter	CXL	Preoperative	1 m	6 m	12 m
UDVA (lgMAR)	Epithelium-on	0.77 ± 0.39	0.62 ± 0.36∗	0.54 ± 0.37∗	0.62 ± 0.37∗
	Epithelium-off	0.67 ± 0.44	0.62 ± 0.45	0.54 ± 0.43∗	0.50 ± 0.44∗
	*P* value	0.394	0.962	0.991	0.289

CDVA (lgMAR)	Epithelium-on	0.20 ± 0.19	0.11 ± 0.14∗	0.06 ± 0.12∗	0.02 ± 0.89∗
	Epithelium-off	0.16 ± 0.13	0.13 ± 0.18	0.09 ± 0.15∗	0.05 ± 0.12∗
	*P* value	0.402	0.633	0.424	0.239

SE (D)	Epithelium-on	−1.58 ± 3.00	−1.32 ± 2.97	−1.97 ± 3.10	−1.73 ± 2.66
	Epithelium-off	−1.81 ± 2.48	−2.06 ± 3.39	−1.61 ± 2.24	−1.68 ± 2.32
	*P* value	0.746	0.436	0.619	0.945

Cylinder (D)	Epithelium-on	−3.19 ± 2.49	−2.58 ± 2.11	−2.64 ± 2.35	−2.66 ± 2.34
	Epithelium-off	−3.54 ± 2.10	−3.26 ± 2.07	−3.09 ± 2.33	−2.79 ± 2.25
	*P* value	0.543	0.167	0.453	0.852

*The difference between pre- and postoperative data was statistically significant (*P* < 0.05).

**Table 4:** Changes in topography features and wavefront aberrations during 12-month follow-up.

Parameter	CXL	Preoperative	1 m	6 m	12 m
Pachymetry (*μ*m)	Epithelium-on	459.50 ± 39.24	445.50 ± 48.92∗	453.50 ± 47.02	458.25 ± 41.09
	Epithelium-off	463.05 ± 31.34	434.35 ± 33.90∗	434.65 ± 31.85∗	450.55 ± 32.14∗
	*P* value	0.625	0.286	0.051	0.273

IRI (*μ*m)	Epithelium-on	34.25 ± 18.57	34.35 ± 18.66	33.65 ± 20.16	35.30 ± 20.09
	Epithelium-off	36.40 ± 13.10	38.05 ± 14.41	34.30 ± 13.91	33.15 ± 13.75
	*P* value	0.658	0.400	0.890	0.618

PE (*μ*m)	Epithelium-on	61.80 ± 24.90	62.25 ± 28.22	60.80 ± 24.11	63.00 ± 31.67
	Epithelium-off	60.65 ± 27.01	59.55 ± 27.30	63.90 ± 25.57	67.35 ± 28.60
	*P* value	0.865	0.717	0.611	0.486

Sim *K*1 (D)	Epithelium-on	47.89 ± 4.46	47.75 ± 4.05	47.83 ± 4.59	47.82 ± 4.10
	Epithelium-off	47.51 ± 2.98	47.52 ± 4.29	47.74 ± 4.43	47.25 ± 3.91
	*P* value	0.695	0.758	0.923	0.544

Sim *K*2 (D)	Epithelium-on	44.29 ± 2.77	44.34 ± 2.77	44.34 ± 2.77	44.47 ± 2.80
	Epithelium-off	44.71 ± 2.98	44.17 ± 3.25	44.17 ± 33.14	44.01 ± 2.97
	*P* value	0.614	0.996	0.776	0.452

*K* max (D)	Epithelium-on	52.68 ± 5.35	52.95 ± 5.38	52.40 ± 5.74	52.78 ± 5.55
	Epithelium-off	53.59 ± 4.72	53.40 ± 5.03	53.58 ± 5.59	53.28 ± 5.18
	*P* value	0.525	0.555	0.620	0.755

RMS: HOAs	Epithelium-on	1.18 ± 0.67	1.20 ± 0.60	1.20 ± 0.71	1.20 ± 0.77
	Epithelium-off	1.15 ± 0.55	1.15 ± 0.51	1.12 ± 0.57	1.07 ± 0.58
	*P* value	0.980	0.496	0.714	0.458

RMS: S3 + 5 + 7	Epithelium-on	1.15 ± 0.65	1.16 ± 0.59	1.12 ± 0.71	1.16 ± 0.76
	Epithelium-off	1.11 ± 0.55	1.03 ± 49	0.97 ± 0.43	0.99 ± 0.52
	*P* value	0.953	0.545	0.769	0.344

*The difference between pre- and postoperative data was statistically significant (*P* < 0.05).
